# Mother-Child Social Cognition Among Multicultural Families in South Korea

**DOI:** 10.3389/fpsyt.2022.883212

**Published:** 2022-06-29

**Authors:** Joohee Lee, Kee-Hong Choi

**Affiliations:** School of Psychology, Korea University, Seoul, South Korea

**Keywords:** multicultural families, marriage immigrant women in Korea, multicultural children, social cognition, emotion recognition, theory of mind

## Abstract

**Objective:**

Despite the rapidly growing number of multicultural families in South Korea, factors influencing parenting and mother-child interactions have not been well-understood. To our knowledge, the present study is the first to have examined how maternal social-cognitive capacity is associated with children's social cognition (e.g., theory of mind and emotion recognition) among multicultural families dwelling in South Korea.

**Methods:**

Forty-seven multicultural mother-child dyads were recruited. The comprehensive measures on social cognition were administered to both the mothers and children, and social functioning and emotion regulation were administered to the children.

**Results:**

A series of hierarchical regressions indicated that mothers' social cognition significantly explained children's ability to recognize static and dynamic emotional expressions, accounting for 27 and 34% of the variance, respectively. Furthermore, mothers' social cognition was significantly correlated to children's social functioning and emotion regulation. However, mothers' social cognition and children's theory of mind were non-significantly related.

**Discussion:**

The current study examined the effects of social cognition of immigrant mothers on their children's socio-emotional development. As the findings indicated an important role of maternal factors (i.e., social cognition) for children's social cognition and their functions, psycho-social approaches (e.g., social cognition parenting education and training) should be incorporated in services for multicultural families.

## Introduction

Since the mid-1990 s, South Korea has rapidly transformed from an ethnically homogeneous country to a multicultural society due to a rapid increase in migrant populations following an influx of international marriage, North Korean refugees, and foreign immigrant workers (1–3). Notably, international marriages in Korea have increased from 4,710 in 1990 to 23,649 in 2019, accounting for 9.9% of the total marriage rate (4). Most of these marriages have occurred between Korean men and foreign women (5, 6), and international marriage-based immigrant women comprise a significant portion of the total number of immigrants in the country (4).

Despite the government's primary focus on immigration and welfare policies to support the settlement of multicultural families in response to the noted phenomenon of migrant feminization, married immigrant women in Korea still face a range of issues, including acculturation, language barriers, social exclusion, financial difficulty, racial discrimination, loneliness, demands of child education, parenting responsibilities, and domestic conflict (7–9). The different sources of stress and the acculturation process simultaneously lower their quality of life and life satisfaction as well as engender psychological difficulties, such as anxiety and depression (6, 10). These accumulative stress factors also influence their parenting styles, passively interacting with their children, developing coercive parenting styles, or showing indifference to their children (11). Hence, foreign-born mothers' poor parenting, lack of interaction, and dysfunctional communication negatively influenced their children's social skills, social competence, and emotional intelligence (12–14), possibly impeding their children's socio-cognitive complications.

Considered newcomers and outsiders, immigrants are less powerful and influential politically while frequently stigmatized negatively by the dominant in-group of the culture. Thus, they must make sense of their new social environment to integrate themselves into the host culture. Their social identities modified by and developed from new environments may influence their social cognition, helping individuals behave appropriately with new cultures and people, thereby facilitating the acculturation process (15). Most marriage immigrant wives arrive in Korea with limited knowledge of the new culture and insufficiency of the Korean language. Although their husbands are usually the primary resources to learn culturally adaptive social skills, their significant differences in ethnic, racial, and cultural backgrounds impede immigrant wives from learning appropriate social skills and knowledge from their husbands. In addition, as others' judgment based on the appearance of minorities may influence their thoughts and feelings (16), the perceived discrimination and social stigma that foreign immigrants often experience may either directly or indirectly impair their social cognition. Indeed, the perceived ethnic discrimination against immigrant mothers and their children could still be pervasive (17–19), and the perceived racial discrimination is likely to increase anger and anxiety, possibly impacting social cognition (20–22). Nagendra et al. (23) found an association between perceived racial discrimination and lower performance in hostility attribution tasks.

Indeed, according to Kim (24), multicultural children showed significantly poorer performance in recognizing and expressing their own emotions compared to mono-cultural children. Although the notion that multicultural family environment is in part tied with mastery of social cognition may be appealing, past research of multicultural families have focused mainly on the various difficulties faced by multicultural families residing in Korea (3, 9, 25, 26) or addressed the relationship between mothers and children in terms of emotion regulation, emotion expressiveness, and emotion knowledge (27–30). To date, no study has specifically explored social-cognitive development among multicultural families.

Social cognition refers to complex cognitive processes of perceiving, interpreting, and processing social information, enabling individuals to behave appropriately in social situations and develop adequate social competence (31, 32). There is a broad consensus among researchers that impaired social cognition in children results in adverse consequences, including externalizing behaviors, conduct problems, and peer rejection (33–36); on the other hand, there is evidence of links between children's social cognition and academic performance, school adjustment, interpersonal relationship, popularity, social competence, and prosocial behaviors (37–43). The current study examined two domains of social cognition: emotion recognition and theory of mind. Prior research has found a correlation between emotion recognition and theory of mind (37, 44–47). Perceiving social information from facial expression, postures, body language, and voice (i.e., emotion recognition) is fundamental in reading and understanding the internal state of others (i.e., theory of mind) (48). That is, emotion recognition and theory of mind are jointly fundamental for enhanced social cognition (49).

Given that family variables have been shown to contribute to individual differences in the rate of social cognition mastery, mothers, as the usual primary caregivers, is an important influence on children's overall social cognition development by providing a responsive and predictable social environment (50, 51), sharing notions of comprehending intentionality and affective states of others, and transferring their social perception to children, and providing sensitive coaching (52–55). Only a few existing studies are suggestive that mothers' social cognition serves as a potential predictor of children's social-cognitive capacity. For example, Castro, Halberstadt (56) found that parents' recognition of children's emotions is positively related to children's recognition of their mothers' emotions, and Ziv and Arbel (53) investigated information processing of mothers and children and found that children resembled their mothers' perceptions of the social world along with strong links between maternal and children's social information processing, especially in the context of a parent-child relationship. Nonetheless, relevant research exploring the relationship between mothers and children's social cognition remains limited, and the past studies that primarily focused on either understanding of emotion or mental states have not rigorously measured various aspects of social cognition.

Besides the influence of maternal social cognition on children's social cognition, mothers' social cognition might also be related to children's social functioning and emotion regulation. As daily interaction with parents foster children's social skills, such as internalizing social rules and expectations, developing communication skills, and constructing a definition of social beings (57), parental belief and attitude toward the social world play an essential role in children's social competence (58). Additionally, evidence concluded that parents' emotion socialization practices, including recognizing emotional expressions of their children and positively responding to children's emotional expressivity, predicted children's advanced skills in regulating their emotions.

### Aims of the Study

Thus, the study's primary purpose was to examine the potential influence of mothers' social cognition on their children's social cognition, social competence, and emotion regulation in a sample of immigrant mothers and their children from 3rd to 6th grade residing in South Korea. We hypothesized that mothers' social cognition would be associated with children's social cognition, namely emotion recognition (Hypothesis 1) and theory of mind (Hypothesis 2); mother's social cognition would be associated with children's social competence (Hypothesis 3) as well as emotion regulation (Hypothesis 4).

## Method

### Participants

Participants were recruited from local multicultural family support centers and online multicultural family communities. Participants included 47 children (*n* = 23 boys, *n* = 24 girls; *M*_*age*_ = 9.81 years, *SD* = 1.26, Range_*age*_ = 8–12 years) and 42 mothers (*M*_*age*_ = 38.79 years, *SD* = 5.04, Range_*age*_ = 31–49 years)—due to the overlapping numbers of mothers as there were five pairs of siblings—from multicultural families residing in South Korea. Child participants were between 3rd and 6th-grade as past research has shown children of this age range to be able to understand and evaluate their own overall social and emotional functioning (29). Children were born between Korean fathers and immigrant mothers. Maternal participants were foreign-born mothers who were international marriage immigrants. Mother's ethnicities were Chinese (40.5%), Vietnamese (16.7%), Mongolian (14.3%), Philippines (14.3%), Japanese (11.9%), and Uzbekistanes (2.4%). Participant demographics are listed in Table 1. Participants were excluded if they met the criteria of neurological disease or a clinical level of mental disorders or if they had difficulty understanding and responding to the questionnaire due to limited Korean proficiency. Data were collected across the country between January and April of 2021.

**Table 1:** Demographic characteristics of participants at baseline.

**Baseline characteristic**	***n***	***%***
**Mother ethnicity**		
Chinese	17	40.5
Vietnamese	7	16.7
Mongolian	6	14.3
Philippines	6	14.3
Japanese	5	11.9
Uzbekistanes	1	2.4
**Mother highest educational level**		
Middle school	1	2.4
High school	11	26.2
Junior college	12	28.6
University	16	38.1
Postgraduate degree	2	4.8
**Mother ocupation**		
Unemployment and housemakers	21	50.0
Agricultural, fishery, and forestry workers, craftworkers, and plant and machine operators	2	4.8
Service and sales workers	3	7.1
Clerical support workers	4	9.5
Professionals	3	7.1
Others	9	21.4
**Family monthly income (unit: 10,000 KWN)**		
Below 100	2	4.8
100–200	8	19.0
200–300	9	21.4
300–400	9	21.4
400–500	10	23.8
Above 500	4	9.5
**Child gender**		
Male	23	48.9
Female	24	51.1

### Procedure

Participants were informed of the study aims and procedures and voluntarily participated and provided informed consent. Each mother-child dyad received monetary compensation for their participation. The study was approved by the local Institutional Review Boards.

Qualtrics, an online survey platform, was used to deliver questionnaires. The survey was conducted in the form of self-reported answers in two different versions, one for mothers and another for children. Participants were given instructions regarding the survey procedure, and then mother and child participants independently completed the sets of questionnaires.

### Measures

#### Emotion Recognition

Two different tasks were used to assess comprehensive emotion recognition ability, recognizing static facial emotional expressions depicted in photographs [i.e., Korean Facial Emotion Identification Task 2; Kim (59)] and dynamic emotional expressions displayed in video clips [i.e., the children's version of the Bell Lysaker Emotion Recognition Task; Lopez, Weinstein (60)].

The Korean Facial Emotion Identification Task 2 (K-FEIT-2) was used to measure the ability to identify seven basic facial emotional expressions (happy, sad, fear, anger, surprise, disgust, no emotion) (59). Participants were presented with 21 randomly ordered Korean facial emotion stimuli and asked to identify the emotion depicted in each stimulus that best applies to one of the seven emotions. The stimuli were kept displayed on the screen until participants responded with no time limit. Potential scores ranged from 0 to 21, with higher scores reflecting greater facial emotion recognition.

The Bell-Lysaker Emotion Recognition Task in Children (BLERT-K) was used to measure emotion recognition, correctly identifying seven emotional states (happy, sad, fear, anger, surprise, disgust, no emotion) based on non-linguistic facial and vocal emotion cues and upper-body movement cues. Participants viewed 8–10 s audiovisual clips of child actors and actresses varying in ethnicity. Three monologs paired with seven emotional states, so there are 21 audiovisual clips. After viewing each video, participants selected the possible seven emotions. The total score ranges from 0 to 21 by calculating the number of correctly identified emotions, and higher scores indicated greater emotion recognition.

#### Theory of Mind

The Social Attribution Task-Multiple Choice (SAT-MC) was used to measure the ability to extract social themes from viewed object motion (61). The SAT-MC comprises a 64-s animation clip of a social drama depicted by a large triangle, small triangle, and small circle. The entire animation was shown before answering questions, and then short segments were presented with multiple-choice questions about the action enacted. Participants needed to answer a total of 19 questions with four possible responses to each. Potential scores ranged from 0 to 19, with higher scores predicting greater theory of mind.

#### Social Functioning

The Social Aptitude Scale (SAS) is an 10-item scale that was used to measure children's social aptitude to understand social and emotional cues rapidly in complex situations to guide socially skilled behavior (e.g., “Easy to chat with, even if it isn't on a topic that specially interests me,” “Aware of what is and isn't appropriate in different social situations”) (62). Participants rated each item on a 5-Likert scale (0 = a lot worse than average; 5 = a lot better than average). The possible score ranges from 0 to 40, with higher scores indicating greater social functioning. Notably, a score of 12 or less indicated difficulties in social functioning and has been associated with autism spectrum disorder (ASD). Internal consistency (α) was 0.90 and 85, respectively, for mothers and children.

#### Emotion Regulation

The Difficulties in Emotion Regulation Scale—Short Form (DERS-SF) is an 18-item self-report scale that was used to measure the magnitude of difficulties in regulating emotion and composes six subscales, including non-acceptance (e.g., “When I'm upset, I become embarrassed for feelings that way”), goals (e.g., “When I'm upset, I have difficulty getting work done”), impulse (e.g., “When I'm upset, I become out of control”), awareness (e.g., “I pay attention to how I feel”), strategies (e.g., “When I'm upset, I believe there is nothing I can do to make myself feel better”), and clarity (e.g., “I have no idea how I am feeling”) (62). Participants rated each item on a 5-Likert scale (1 = almost never; 5 = almost always), and possible scores ranged from 18 to 90, with higher scores indicating severe emotional dysregulation. For mothers and children, internal consistency (α) was 90 and 0.84, respectively.

#### Acculturative Stress

The Acculturative Stress Scale (ASS) is a 36-item self-report scale that was used to measure the level of stress associated with the immigration and acculturation process among multicultural immigrant mothers (63). The scale has seven subscales: perceived discrimination (e.g., “I feel that I receive unequal treatment”), perceived hatred/rejection (e.g., “People show hatred toward me verbally”), fear (e.g., “I feel insecure here”), cultural shock/stress (e.g., “I feel uncomfortable to adjust to new foods”), guilt (e.g., “I feel guilty to leave my family and friends behind”), and others (e.g., “It hurts when people don't understand my cultural values”). Participants rated each item on a 5-point Likert scale (1 = strongly disagree; 5 = strongly agree). Potential scores ranged from 36 to 180, with higher scores indicating higher levels of acculturative stress. Internal consistency (α) was 0.96.

The modified ASS is a 17-item self-report scale that was used to measure specific acculturative stressors of multicultural children (64). The original ASS includes a set of subscales that are irrelevant to children born into multicultural families, such as homesickness (e.g., homesickness bothers me), cultural shock (e.g., I feel uncomfortable to adjust to new cultural values), guilty (e.g., I feel guilty to leave my family and friends behind). Kim and Kim (64) designed the modified version of the ASS for multicultural children based on a focus group interview. The scale consists of three subscales: perceived discrimination (e.g., “I am denied what I deserve”), Korean language fluency (e.g., “It is hard for me to do homework due to my limited Korean”), and alienation [e.g., “I don't feel sense of belonging (community) here”]. Participants rated on a 5-point Likert scale (1 = strongly disagree; 5 = strongly agree). Potential scores ranged from 17 to 85, with higher scores indicating higher levels of severe acculturative stress. The internal consistency (α) was 0.86.

#### Data Analysis

Data analysis was conducted using SPSS 25.0. A series of Pearson's correlations and hierarchical regressions were used to examine the relationship between mothers' social cognition and children's social cognition, social functioning, and emotion regulation. Each aspect of social cognition, social functioning, and emotion regulation was analyzed in a separate model.

Any significant differences in demographic characteristics, including family income, mother ethnicity, mother education, mother occupation, child gender, and child grade, were examined. Only children's gender showed a significant difference in children's theory of mind, which was controlled in the model.

In addition, mothers' age and Korean language fluency of both mothers and children were considered in the regression model to control the potential confound effects. Given that emotion regulation is correlated with acculturative stress among multicultural families (65), acculturative stress of mother and children was further added to the first step of emotion regulation model analysis. Then, to test the collective influence of mothers' social cognition on children's social-cognitive and -emotional development, mothers' theory of mind and emotion recognition were entered in the second and last step. Five separate hierarchical regression analyses were performed to examine the predictors of children's social cognition (i.e., emotion recognition and theory of mind), social competence, and emotion regulation. Noted that emotion recognition was assessed by two different tasks, recognizing static facial emotional expressions depicted in photographs and recognizing dynamic emotional expressions displayed in video clips. Each step was evaluated for statistical significance, and individual variables were assessed within each step.

As the tolerance statistic is above the recommended 0.2 level and the VIF was below the proposed critical value of 10 across all models (66), no multicollinearity occurred, indicating that the estimated βs were well-established in the following regression models.

## Results

### Descriptive and Correlational Analysis

Table 2 presents the descriptive statistics of the key variables and their bivariate correlations. Mothers' theory of mind was significantly and positively related to their recognition of static emotions (*r* = 0.52, *p* < 0.01) and negatively related to children's emotion dysregulation (*r* = −0.45, *p* < 0.01). Mothers' recognition of dynamic emotional expressions was significantly positively related to their recognition of static emotions (*r* = 0.50, *p* < 0.01), children's recognition of dynamic emotions (*r* = 0.49, *p* < 0.01), and children's social competence (*r* = 0.48, *p* < 0.01), and was negatively related to children's emotion dysregulation (*r* = −0.42, *p* < 0.01). Mothers' recognition of static emotions was significantly positively related to children's theory of mind (*r* = 0.34, *p* < 0.05), dynamic emotion recognition (*r* = 0.42, *p* < 0.01), and static emotion recognition (*r* = 0.45, *p* < 0.01). Mothers' acculturative stress was significantly negatively related to children's static emotion recognition (*r* = −0.30, *p* < 0.05).

**Table 2:** Descriptive statistics and correlations for study variables.

**Variables**	**M**	**SD**	**1**	**2**	**3**	**4**	**5**	**6**	**7**	**8**	**9**	**10**
1. Mother SAT	13.11	4.00	1									
2. Mother BLERT	16.30	2.70	0.24	1								
3. Mother K-FEIT	18.13	1.88	0.52^**^	0.50^**^	1							
4. Mother ASS	79.98	25.60	−0.19	−0.11	−0.23	1						
5. Child SAT	14.34	3.99	0.27	0.12	0.34^*^	−0.02	1					
6. Child BLERT	16.62	2.37	0.14	0.49^**^	0.42^**^	−0.22	0.30^*^	1				
7. Child K-FEIT	16.26	2.21	0.19	0.13	0.45^**^	−0.30^*^	0.33^*^	0.35^*^	1			
8. Child ASS	25.64	7.92	0.02	−0.09	0.08	0.02	−0.03	−0.21	−0.23	1		
9. Child DERS	41.45	11.58	−0.45^**^	−0.42^**^	−0.25	0.12	−0.15	−0.23	−0.34^*^	0.40^**^	1	
10. Child SAS	20.81	5.95	0.22	0.48^**^	0.29	−0.29	0.18	0.47^**^	0.38^**^	−0.26	−0.53^**^	1

* *p < 0.05*;

** *p < 0.01. SAT-MC, Social Attribution Task-Multiple Choice; BLERT-K, Bell-Lysaker Emotion Recognition Task in Children; K-FEIT, Korean Facial Emotion Identification Task; ASS, Acculturative Stress Scale; DERS, Difficulties in Emotion Regulation Scale; SAS, Social Aptitude Scale*.

Children's theory of mind was significantly and positively related to recognizing dynamic emotions (*r* = 0.30 *p* < 0.05) and static emotions (*r* = 0.33 *p* < 0.05). Children's recognition of dynamic emotions was significantly positively related to their recognition of static emotions (*r* = 0.35 *p* < 0.05) and social competence (*r* = 0.47 *p* < 0.01). Children's recognition of static emotions showed a significant negative correlation with their emotional dysregulation (*r* = −0.34 *p* < 0.05) and a positive correlation with their social competence (*r* = 0.38 *p* < 0.01). Children's acculturative stress was significantly positively correlated with emotional dysregulation (*r* = 0.40 *p* < 0.01), and there was a significant negative relationship between children's emotional dysregulation and social competence (*r* = −0.53, *p* < 0.01).

#### Mother's Social Cognition in Relation to Children's Social Cognition

##### Emotion Recognition

In the Model 1, the overall model was significant for children's ability to recognize static facial expressions depicted in photographs [*F*_(6,40)_ = 2.49, *p* < 0.05], and the predictors explained 27% of the variance. Mothers' recognition of static emotions was significantly and positively related to children's recognition of static emotions over and above the potential confounding variables entered into preceding steps (β = 0.63, *p* < 0.01) (Table 3).

**Table 3:** Hierarchical regression model predicting children's social cognition.

	**Dependent Variable**								
**Variable**	**Child static emotion recognition**			**Child dynamic emotion recognition**			**Child theory of mind**		
	**B**	**β**	***t*(p)**	**B**	**β**	***t*(p)**	**B**	**β**	***t*(p)**
**Step 1**									
Mother age	−0.06	−0.13	−0.84	−0.02	−0.04	−0.29	0.08	0.10	0.72
Mother Korean language	0.10	0.07	0.48	0.27	0.19	1.22	0.21	0.09	0.61
Child Korean language	−0.43	−0.13	−0.83	0.73	0.20	1.32	0.73	0.12	0.84
Child gender (1 = male)							3.27	0.41	2.95
*R*^2^ (adj.)	0.04 (−0.02)			0.06 (−0.01)			0.20 (0.12)		
*F(p)*	0.65			0.88			2.63		
**Step 2**									
Mother age	−0.08	−0.18	−1.18	−0.10	−0.20	−1.38	0.13	0.17	1.15
Mother Korean language	−0.17	−0.13	−0.86	0.06	0.04	0.30	−0.04	−0.02	−0.12
Child Korean language	−0.42	−1.13	−0.90	0.64	0.18	1.34	0.72	0.12	0.89
Mother theory of mind	−0.10	−0.17	−1.00	−0.11	−0.18	−1.07	3.64	0.46	3.44
Mother dynamic emotion recognition	−0.06	−0.08	−0.46	0.35	0.40	2.60^*^	0.23	0.23	1.39
Mother static emotion recognition	0.74	0.63	3.22^**^	0.40	0.32	1.72	−0.04	−0.03	−0.18
*R*^2^ (adj.)	0.27 (0.16)			0.34 (0.24)			0.36 (0.24)		
*F(p)*	2.49^*^			3.44^**^			3.12		

* *p < 0.05*;

** *p < 0.01*.

In the Model 2, the addition of the mothers' social cognition in the final step resulted in significant change for children's ability to recognize dynamic emotional expressions and accounted for 34% of the variance [*F*_(6,40)_ = 3.44, *p* < 0.01]. Only mothers' ability to recognize dynamic emotions was uniquely related to children's such ability (β = 0.40, *p* < 0.05) (Table 3).

##### Theory of Mind

In contrast to emotion recognition, the overall model was not significant. Neither mother's emotion recognition nor theory of mind were significant predictors of children's theory of mind when included in the regression model (Table 3).

#### Mother's Social Cognition in Relation to Children's Social Functioning

The overall model was significant with variables accounting 29% of the variance in children's social functioning [*F*_(6,40)_ = 2.69, *p* < 0.05]. Mother's emotion recognition of dynamic emotional expressions significantly predicted children's social functioning (β = 0.44, *p* < 0.01) (Table 4).

**Table 4:** Hierarchical regression model predicting children's social functioning and emotion regulation.

	**Dependent Variable**					
**Variable**	**Child social functioning**			**Child emotion regulation**		
	**B**	**β**	***t*(p)**	**B**	**β**	***t*(p)**
**Step 1**						
Mother age	0.24	0.15	1.00	0.07	0.03	0.22
Mother Korean language	1.05	0.22	1.44	0.82	0.12	0.80
Child Korean language	1.04	0.09	0.57	−0.75	−0.04	−0.29
Mother acculturative stress				0.06	0.13	0.91
Child acculturative stress				0.78	0.40	2.83
*R*^2^ (adj.)	0.08 (0.01)			0.19 (0.10)		
*F(p)*	1.18			1.97		
**Step 2**						
Mother age	0.17	0.11	0.71	0.00	0.00	0.01
Mother Korean language	0.98	0.21	1.37	0.99	0.14	1.08
Child Korean language	0.68	0.06	0.41	−0.08	−0.00	−0.04
Mother acculturative stress				0.01	0.03	0.26
Child acculturative stress				0.73	0.37	3.11
Mother theory of mind	0.41	0.21	1.19	−1.17	−0.40	−2.66^*^
Mother dynamic emotion recognition	1.29	0.44	2.73^**^	−1.39	−0.33	−2.27^*^
Mother static emotion recognition	−0.48	−0.12	−0.60	0.30	0.05	0.28
*R*^2^ (adj.)	0.29 (0.18)			0.48 (0.37)		
*F(p)*	2.69^*^			4.31^**^		

* *p < 0.05*;

** *p < 0.01*.

#### Mother's Social Cognition in Relation to Children's Emotion Regulation

The results of the regression indicated the predictors explained 48% of the variance [*F*_(8,38)_ = 4.31, *p* < 0.01]. It was found that mothers' recognition of dynamic emotional expressions contributed significantly to the explanation of children's emotion regulation (β = −0.33, *p* < 0.05), as did mothers' theory of mind (β = −0.40, *p* < 0.05) (Table 4).

## Discussion

Over the recent years, investigating prospective familial and parental factors that contribute to children's socio-emotional and socio-cognitive development has increasingly become a crucial task. The current study was the first to investigate how influential mothers' social cognition was on children's social cognition and whether mothers' social cognition was linked to children's social competence and emotion regulation using a sample of married immigrant mothers and their children from multicultural families. As predicted, mothers' general social cognition skills—their emotion recognition and theory of mind—positively related to their children's emotion recognition, social competence, and emotion regulation even when controlling for demographic characteristics, family-level characteristics, and potential effects of Korean language proficiency and acculturative stress. However, neither component of the mothers' emotion recognition and theory of mind significantly correlated with their children's theory of mind. The main findings in this study are discussed in greater detail below.

First, according to the first hypothesis, we found that mothers' and children's emotion recognition were positively correlated. Precisely, the mother's ability to recognize static emotions did, in turn, predict their children's recognition of static emotional expressions, while mothers' recognition of dynamic emotions was related to children's recognition of dynamic emotional expression. The strong reciprocal relationship between mothers' and children's dynamic emotion recognition and between mothers' and children's static emotion recognition could be explained by Common Method Bias (CMB), which occurs when the independent and dependent variables are measured using the same method, possibly inflating their relationship (67). In addition, the associations between mothers' and children's emotion recognition add accumulating evidence to the finding of a previous study in which parents' ability to recognize children's emotions was uniquely related to children's recognition of their parents' emotions (56). Earlier research has shown that parents' supportive responsiveness to their children's emotional expression helped children to access and learn about their own and other people's feelings in a range of emotional and social contexts and consequently acquire a more advanced understanding of emotion (68–70). These results suggest that mothers first need to be competent in detecting and labeling diverse emotions to respond appropriately to their children's emotional expressions.

Second, contrary to the second hypothesis, we did not find a significant association between mothers' social cognition and children's theory of mind. Children's theory of mind ability was related to neither mothers' emotion recognition nor theory of mind. This result is not consistent with Ziv and Arbel (53), who concluded that mothers' ability to understand others' intentions (i.e., social information processing) affected children's social information processing patterns through mothers' parenting styles. It has been argued that when children acknowledge that their mothers correctly mirror and reflect their emotional states, children are able to relax and feel more secure, ultimately enhancing their mental capacities to focus on psychosocial processes and facilitating their understanding of mental states of both others and themselves (55).

The explanation for a failure to observe a correlation between mothers' social cognition and children's theory of mind is likely that we did not consider maternal practices in terms of parenting that would have more influence on children's theory of mind growth. For example, given that mothers' preference of affective and mentalist conversation (71–73), mental state language (74), and elaborative discourse (54) has been proven to have a significant impact on children's theory of mind performance. These findings strongly suggest that mothers' reference to the relationship among emotions, beliefs, and behaviors and mother's use of mental state terms that underscore different perspectives help children learn the subjective nature of mental state, which eventually improves children's understanding of themselves and others (75). In addition, mothers' social cognition may not correctly reflect mothers' sensitivity to their children's mental states. Especially for multicultural families, immigrant mother's different cultural backgrounds and language barriers might negatively affect mothers' engagement in practices of responding to their children's mental state and talking about feeling states in a proper and timely manner (76, 77), regardless of mothers' competence in theory of mind. Future research should examine both mothers' social cognition ability and mother-child mental state conversation to clarify if mothers' mental state talk mediates the relationship between mothers' and children's social cognition or independently affects children's theory of mind development.

Third, concerning the third hypothesis, there was a significant and positive correlation between mothers' emotion recognition and children's social competence. Mothers' ability to recognize dynamic emotional expressions showed a significant relationship with children's social competence, while mothers' ability to recognize static facial expressions did not. This discrepancy could have occurred simply because of the task at hand. Recognizing emotional expressions with a complex set of affective information transmitted in prosody or body posture has a greater verisimilitude with real-life events, so it assesses emotion recognition ability more correctly than the task of recognizing static emotional expressions depicted in photos.

It has been well-established that the ability to recognize basic emotions in others' emotional expressions impacts behavioral and emotional reactions in social contexts (78), suggesting a close tie between emotion recognition and social competence. When children encounter new social situations, they access their database constructed through previous experience regarding others' feelings, thoughts, and intentions to behave appropriately, meeting general social expectations (79). Particularly in children, the database was mainly built from parental guidance (80), and therefore the ability to identify emotions and infer complex mental states is necessary for parents to teach their children socially adaptable skills. Further, mothers' awareness of children's emotions can predict their interpersonal relationships with their children; several studies have emphasized the effect of quality of attachment and the mother-child relationship on children's social functioning (81–83). Children may develop more sophisticated social functioning by building a higher quality of mother-child relationships. Thus, using the additional information (e.g., attachment and mother-child relationships) in future studies is recommended to disclose possible confounding variables explaining variance in association between mothers' social cognition and children's social competence.

Finally, the results did support the fourth hypothesis, showing that mothers' emotion recognition and theory of mind concurrently affected children's ability to manage emotions. Emotion regulation depends on social-cognitive input derived from observable and inferential information sources, implying that social cognition directly influences the regulation of emotional responses (84). The association between emotion regulation and social cognition is particularly salient in clinical conditions where impaired emotion recognition is typically reported in several mood disorders relevant to severe emotion dysregulation, including bipolar disorder and depression (85–88). Our results provided further specific findings that, in addition to children's own social cognition capacity, mothers' social cognition plays a key role in amplifying children's successful implementation of emotion regulation strategies.

Given that numerous research emphasized emotion coaching as a predictor of child emotion regulation (89–93), mothers' social cognition may relate to children's emotion regulation as emotion coaching demands social-cognitive skills to some degree. Mothers first must recognize and understand children's affective and cognitive states to validate and acknowledge their feelings and provide appropriate emotional coaching (90). Thus, it makes sense that mothers skilled in emotion recognition and theory of mind can respond to their children's emotional expressions and behaviors at a satisfactory level, thereby improving children's process of managing their emotions.

Intriguingly, the mothers' recognition of dynamic emotional expression and theory of mind was lower than that of children. Given that individuals are generally more likely to accurately recognize their vocal emotions from their native language (94), the dynamic emotion recognition task re-recorded in the Korean language might negatively impact the mothers' performance. Although the social attribution test—multiple choice (SAT-MC) employed in the current study is non-verbal using geometric shapes and is thus less culturally designed than other social cognition measures, the immigrant mothers performed slightly worse on the theory of mind task than the average score from a healthy undergraduate student sample from the United States and South Korea, whose mean scores were 16.53 and 14.98, respectively (95, 96). In contrast, the multicultural children showed the adult level of theory of mind, which could be explained by the fact that dual language exposure from an early age has been suggested to benefit the development of theory of mind (97). Nonetheless, little research has examined the factors influencing theory of mind development among minorities and multicultural families. As stress has negatively affected emotion decoding performance (98), immigrant mothers' extensive, diverse stressors in acculturation, rearing children, and family conflicts may lower the performance of emotion recognition and theory of mind. Therefore, future research should identify the specific variables moderating the social cognition of immigrants to provide a clearer understanding of the relationship between mothers and their children's social cognition within minority families.

Although these findings expand our understanding of the role of mothers' social cognition in children's social-cognitive and -emotional development, several limitations must be acknowledged. First, the study only analyzed social cognition and emotional outcomes of mother and child subjects under the assumption that mothers are likely to spend more time fostering their children than fathers. An abundance of literature has suggested that the quality of the father-child relationship also significantly impacts children's psychological wellbeing (99–101). Moreover, fathers and mothers play distinct roles in the development of children's socio-cognitive competence. Mazzone and Nader-Grosbois (73) found that mothers' emotion-related conversations influenced children's theory of mind ability, while fathers' emotion-related conversations influenced children's emotion regulation. LaBounty, Wellman (102) demonstrated that mothers' use of internal state talk predicted children's emotional understanding, and fathers' use of internal state talk predicted children's theory of mind. In addition, parental socio-emotional practices could vary with children's gender (103, 104). Hence, future efforts should examine the role of gender-related factors in both parents and children in terms of children's socio-emotional development. Another limitation of the study was that the sample size was relatively small to run more advanced statistical techniques, such as a structural equation modeling analysis. This present study used a cross-sectional design which prevented any inference regarding the cause or bidirectional relations among the study variables. Future research should, then, replicate the results by conducting longitudinal studies to illuminate further the role that mothers' social cognition plays in developing children's social cognition, social competency, and emotion regulation—and whether such variables affect each other bi-directionally over time. Lastly, the present research specifically focused on middle childhood. To fully understand the growth pathway of social cognition, studying only a particular age group, such as middle childhood, could limit the holistic understanding of cognitive development across children and adolescents. Thus, further replications with a broader age group are required to ensure the generalizability of the findings.

Despite these limitations, the current results have important implications for the role of social cognition in children's socio-emotional development. To the best of our knowledge, the present study is the first to reveal that mothers' social cognition relates to children's emotion recognition, emotion regulation, and social competence among multicultural families, suggesting that multicultural children advance their social-cognitive capability in relation to foreign-born mothers' social cognition. Consequently, support meant to improve immigrant women's social-cognitive performance could be a crucial factor in developing children's social cognition skills, ultimately facilitating social competence and emotion regulation. To date, past research on multicultural families has primarily targeted their emotion-related deficits, maladjustment, and acculturation stress, and much less attention has been paid to their social cognition. In this context, the present study conveys an optimistic message: social cognition can act as a beneficial resource that promotes children's development. Furthermore, this type of research not only provides directions for future studies investigating the social cognition of multicultural families, but it also highlights the need for special assistance or appropriate intervention to train and equip multicultural families with the adaptive level of social cognition. Padilla and Perez (15) pointed out that immigrant populations have difficulty assimilating new information and social rules of a new country into the existing schema acquired from their mother countries, hindering their primary social cognitive performance and negatively impacting their children's socio-emotional growth. In this respect, we expect social cognitive training to guide immigrant mothers to think and behave within appropriate cultural values and norms, contributing to normalizing immigrants' social cognitive performance. This could help them adjust to new societies and pass down adaptive social cognitive and emotional strategies to their children.

## Data Availability Statement

The raw data supporting the conclusions of this article will be made available by the authors, without undue reservation.

## Ethics Statement

The studies involving human participants were reviewed and approved by Korea University Institutional Review Board. Written informed consent to participate in this study was provided by the participants' legal guardian/next of kin.

## Author Contributions

JL and K-HC: conception, design of the study, and revision of the manuscript. JL: data collection, data analysis, data interpretation, and drafting the manuscript. All authors read and approved the final version of the manuscript.

## Funding

This work was supported by the Ministry of Education of the Republic of Korea, the National Research Foundation of Korea (NRF-2017S1A5B6053101), and grant funded by the Korea government (MSIT) (No. NRF-2020R1A2C2099665).

## Conflict of Interest

The authors declare that the research was conducted in the absence of any commercial or financial relationships that could be construed as a potential conflict of interest.

## Publisher's Note

All claims expressed in this article are solely those of the authors and do not necessarily represent those of their affiliated organizations, or those of the publisher, the editors and the reviewers. Any product that may be evaluated in this article, or claim that may be made by its manufacturer, is not guaranteed or endorsed by the publisher.
